# Protocol of the DENIM study: a Delphi-procedure on the identification of trauma patients in need of care by physician-staffed Mobile Medical Teams in the Netherlands

**DOI:** 10.1186/s13049-015-0089-z

**Published:** 2015-02-08

**Authors:** Annelieke Maria Karien Harmsen, Leo Maria George Geeraedts, Georgios Fredericus Giannakopoulos, Maartje Terra, Herman Martinus Timotheus Christiaans, Lidwine Brigitta Mokkink, Frank Willem Bloemers

**Affiliations:** Department of Surgery, VU University Medical Centre Amsterdam, P.O. Box 7057, 1081 HV Amsterdam, The Netherlands; Department of Surgery Slotervaart Hospital, Amsterdam, The Netherlands; Department of Anaesthesiology, VU University Medical Centre Amsterdam, Amsterdam, The Netherlands; Department of Epidemiology and Biostatistics, VU University Medical Centre Amsterdam, Amsterdam, The Netherlands

**Keywords:** DENIM, Delphi, Protocol, Dispatch, Trauma, Mobile Medical Team, Helicopter, Ambulance

## Abstract

**Background:**

In The Netherlands, standard prehospital trauma care is provided by emergency medical services and can be supplemented with advanced trauma care by Mobile Medical Teams. Due to observed over and undertriage in the dispatch of the Mobile Medical Team for major trauma patients, the accuracy of the dispatch criteria has been disputed. In order to obtain recommendations to invigorate the dispatch criteria, this study aimed at reaching consensus in expert opinion on the question; which acute trauma patient is in need of care by a Mobile Medical Team? In this paper we describe the protocol of the DENIM study (a Delphi-procedure on the identification of prehospital trauma patients in need of care by Mobile Medical Teams).

**Methods:**

A national three round digital Delphi study will be conducted to reach consensus. Literature was explored for relevant topics. After agreement on the themes of interest, the steering committee will construct questions for the first round. In total, 120 panellists with the following backgrounds; Mobile Medical Team physicians and nurses, trauma surgeons, ambulance nurses, emergency medical operators will be invited to participate. Group opinion will be fed back between each round that follows, allowing the panellists to revise their previous opinions and so, converge towards group consensus.

**Discussion:**

Successful prehospital treatment of trauma patients greatly depends on the autonomous decisions made by the different professionals along the chain of prehospital trauma care. Trauma patients in need of care by the Mobile Medical Team need to be identified by those professionals in order to invigorate deployment criteria and improve trauma care. The Delphi technique is used because it allows for group consensus to be reached in a systematic and anonymous fashion amongst experts in the field of trauma care. The anonymous nature of the Delphi allows all experts to state their opinion whilst eliminating the bias of dominant and/or hierarchical individuals on group opinion.

## Background

**Figure 1:**
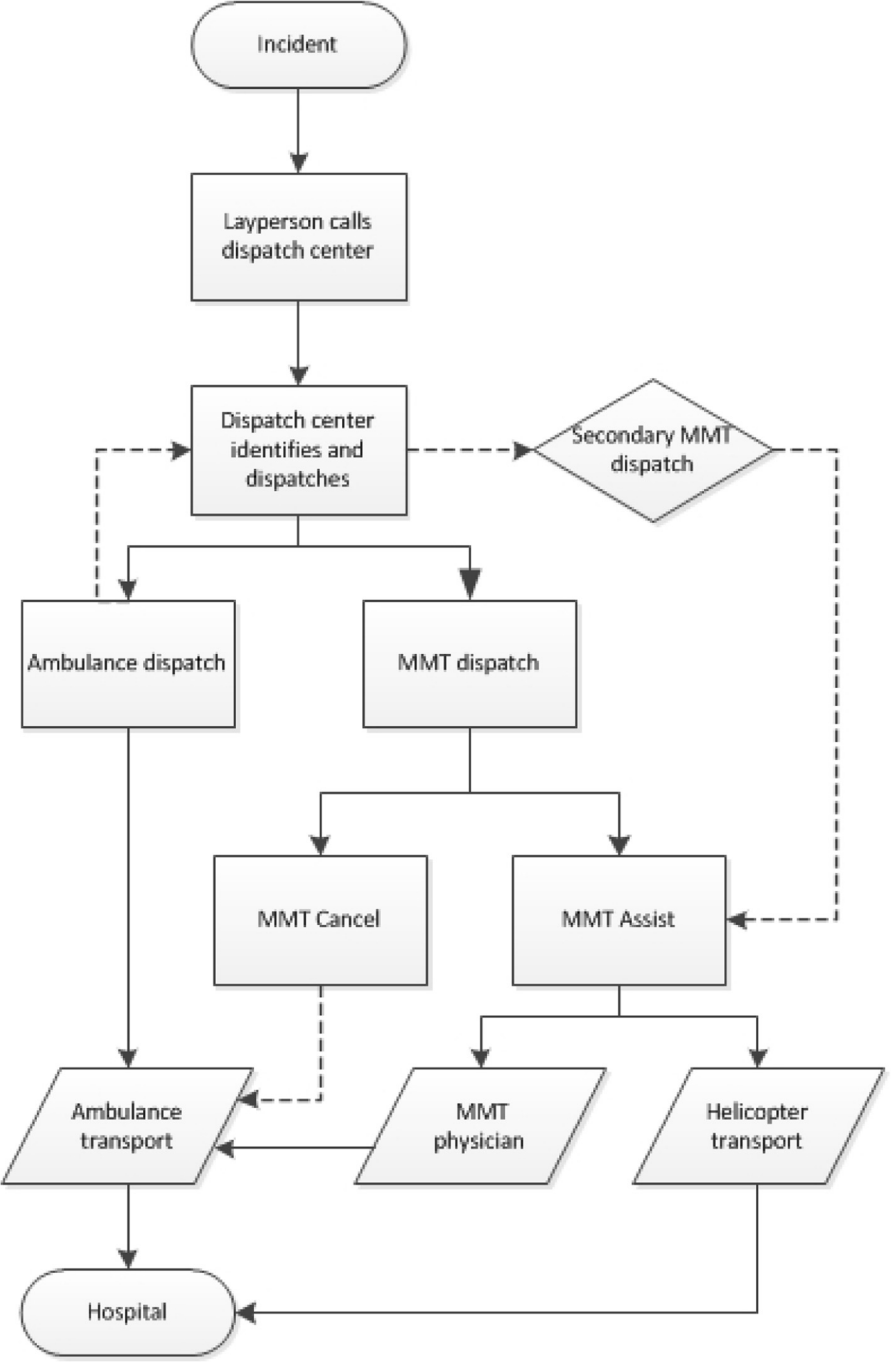
**Schedule of ambulance and MMT dispatch; MMT: Mobile Medical Team.**

In The Netherlands standard prehospital trauma care is provided by emergency medical services (EMS). All EMS care providers are highly trained and registered nurses with certification in either anaesthesia, intensive care, cardiac care or emergency care and additional training in prehospital trauma life support [1,2]. In order to enhance prehospital care for the severely injured patient in The Netherlands, the Mobile Medical Team (MMT) was introduced in 1995 and was extended by night flight coverage in 2006 [3]. Nowadays, The Netherlands is covered by four MMTs that are stand-by 24/7 and have the availability of either helicopter or road ambulance transportation. A MMT rapidly delivers advanced trauma life support to the trauma patient in the out-of hospital setting. A Dutch MMT consists of either a specialized anaesthesiologist or trauma surgeon and a specialized trauma nurse with at least five years of working experience in the Emergency Room or at the EMS. The MMT supplements the prehospital trauma life support performed by the EMS with advanced trauma care according to, but also beyond Advanced Trauma Life Support. Procedures performed are, amongst others, rapid sequence intubation, advanced pain management, the administration of inotropes, vasopressors and other medication. Moreover, a MMT can perform invasive surgical interventions such as surgical airway, intercostal drainage, splinting, thoracotomy and advanced haemorrhage control. The primary objective of a MMT is swift transport of advanced trauma care to the injured trauma patient in order to perform early life saving interventions. MMTs are mainly transported by helicopter (69%), but also by road [4]. In most of the cases the MMT physician accompanies the patient to the hospital in the EMS road ambulance and in 5-20% of the cases the trauma patient is transported by the MMT in the helicopter. At the scene, the MMT physician is responsible for the prehospital logistical process. The MMT physician decides on the type and order of treatment as well as to which hospital the trauma patient should be transported, based on their knowledge and experience as specialists in trauma care augmented by their frequent exposure to specific situations and patient conditions. Though MMT care has been implemented for several years now, deployment of the teams could be more efficient. A study by Giannakopoulos et al. showed an overtriage rate of 26% for one of the Dutch MMT’s [4]. Another study in the same cohort of dispatches showed that 21% of all cancellations of this MMT concerned major trauma patients [5]. This may be interpreted as undertriage, as this patient category is thought to benefit most by the prehospital assistance of the MMT. Differences in interpretation and application of the MMT dispatch and cancellation criteria by emergency medical personnel may be an underlying cause. Several reasons for this phenomenon can be listed such as regional differences in working culture (and familiarity with MMT care), professional autonomy of care takers in all involved disciplines (adherence to guidelines) and a difference in trauma-related knowledge and/or exposure. Current dispatch criteria are active since June 2013 and based on two national ambulance protocols and a study by Ringburg et al. reviewing dispatch criteria [6,7]. Key topics of the current criteria are shown in Table 1. Based on these assumptions, the nature of the incident, location and time of transport appear to be of secondary importance. In the available literature, many articles describe research in the U.S. paramedic based EMS-setting or in the German physician based prehospital system-setting. The findings of this research cannot simply be compared or extrapolated to the Dutch hybrid (EMS and MMT) prehospital system [4,6]. Current dispatch criteria are mainly based on level 4 evidence (expert opinion and experience) [8], with the exception of loss of consciousness which has been proven a reliable and validated parameter for Helicopter Emergency Medical Services (HEMS) dispatch [6]. In the event of a severe trauma, emergency operators in the dispatch centre deploy the MMT simultaneously with the EMS ambulance crew (dispatch sequence is displayed in Figure 1). The decision for dispatch is done based on information handed to the operator by a layperson. Because this information can be incomplete or incorrect the dispatch centres handles a low activation threshold for dispatch to minimise undertriage. The National Institute for Public Health and the Environment (RIVM) report on distinct differences in the absolute numbers of dispatches between the four Dutch MMTs [9]. Several possible reasons for the occurrence of these differences are suggested. Firstly, the RIVM report shows that the greater the geographical distance between the dispatch centre and the MMT-base, the less likely emergency operators are to deploy the MMT. Organisational and management factors such as limited or insufficient protocol implementation in the dispatch centre may be of influence. Finally, sociocultural aspects may play a role; including inexperience, biased working culture, individual attitudes, poor communication and levels of training of both ambulance and MMT personnel [9]. The DENIM study (‘*DELphi studie in Nederland naar de Inzet van het MMT’* Delphi study in the Netherlands on the dispatch of the Mobile Medical Team) aims at reaching consensus in expert opinion on the question; ‘which trauma patient deserves the advanced care provided by a MMT?’. This consensus can then be used to invigorate MMT dispatch criteria in the future. The objective of this paper is to describe the design of the DENIM study.

**Table 1:** **Fundamental tenets of the current MMT dispatch criteria**

1.	For the dispatch of medical personal the condition of the patient is determinative.
2.	A patient with unstable vital parameters has the right to receive maximal medical care.
3.	The type of care delivered is determined by the severity of the deviation in vital parameters.
4.	MMT care is an extension of prehospital medical care by ambulance personnel
5.	MMT care focuses mainly on stabilisation of the vital parameters

MMT: Mobile Medical Team.

## Method

### The Delphi technique

The DENIM study uses the Delphi technique, which was initially developed in the 1950’s by the RAND Corporation. This intelligence *think tank* designed the Delphi for use on complex problems that exceed the analytical capabilities of a single person and need to be addressed by a group of experts [10]. The Delphi technique is a structured approach of anonymous debating to generate discussion and converge toward group consensus. This is achieved through a series of rounds in which experts have to answer questionnaires [11]. The responses are then analysed and anonymously fed back to the panellists in a subsequent questionnaire. The feedback report entails an anonymous summary of the panellist’s group opinion with the associated argumentation, in order to encourage the panellist to revise their previous opinions in light of the replies of the other panellists [12]. This process may be repeated any number of times, it is thought that the group opinion will evolve towards a consensus. It is of scientific value because it can lead to an agreed set of recommendations to guidelines [13]. This study was approved by the Medical Ethics Review Committee of the VU University Medical Center.

### Literature search

To construct the questionnaire, literature was reviewed to derive information on current dispatch criteria, conditions and terms of establishing dispatch criteria in other prehospital settings, information on sensitivity of separate criteria to identify major trauma patients and other factors of influence on dispatch of the MMT. An electronic search in PubMed, EMBASE.com and The Cochrane Library (via Wiley) was conducted. PubMed was searched using a combination of medical subject headings (Mesh) and keywords (Web appendix 1). We applied a language restriction; English, German and Dutch articles were included. The separate results from MEDLINE, Embase and the Cochrane library were checked for duplicate articles. All articles were reviewed and assessed for suitability based on title and abstract by two independent reviewers (AH and GG). Inclusion criteria were articles reporting on (1) trauma patients and (2) dispatch and/or cancel criteria for a MMT, HEMS or physician-staffed EMS. Articles reporting (1) solely on paramedic dispatch criteria, (2) articles with no full-text available, (3) comments to other papers, (4) and editorials were excluded. Discrepancies were resolved by consensus.

### Delphi steering committee

The steering committee comprises of members with an occupational background within the field of prehospital and/or inhospital trauma care. The expertness characteristics of the team include anaesthesiology, trauma surgery, general surgery and MMT. Furthermore the steering committee is strengthened by a member (LM) with expertise in performing Delphi studies. The steering committee, consisting of all authors of this paper (except LM), will decide on which topics are relevant to include in the Delphi study and the type and manner of questioning. Three members of the steering committee will structure the questionnaire. The preliminary questionnaire will be send to all members of the steering committee for final comments and adjustments. The steering committee will furthermore undertake the analysis of the data, composing of the feedback documents, generating the subsequent questionnaires and overall supervision and general management of the Delphi process. The steering committee will prepare, supervises and monitor all Delphi rounds and will not take part as panel members.

### Delphi expert panel

Professionals within the field of prehospital and the inhospital trauma patient care will be recruited to participate if they had the following background: MMT physicians and nurses, trauma surgeons, ambulance paramedics, emergency medical operators. Experts will be identified through nomination by steering committee members using their networks, by contacting the Dutch societies for trauma surgeons, anaesthesiology and paramedics. Furthermore the Dutch consortium for emergency medical operators will be approached as well as the chief doctors of the four Dutch MMTs. Subsequently, a heterogenic expert panel will be created, in which all the disciplines involved in prehospital trauma care will be represented [14]. There are no clear numbers on adequate panel size for a Delphi study [11]. Therefore, we arbitrarily decided that a panel had to consist of at least 10 experts per category of expert background to be adequate, a combined total of approximately 50 members. In previous Delphi studies the maximum response rate is up to 70% for the first round and 50% will suffice to complete the entire survey [15,16]. Therefore approximately 120 panellists will be solicited to participate. All eligible panellists will be contacted via email, introducing the Delphi study and asking them to participate. Background information on the aim and course of the study will be given. Experts who do not respond will be reminded twice. When less than 70 panellists agree to participate, 50 more panellists will be invited while keeping in mind that all different disciplines need to be represented equally to ensure heterogeneity. Panellists will remain anonymous throughout the entire study. The research coordinator has access to panellist’s information for logistical reasons.

### The Delphi structure

**Figure 2:**
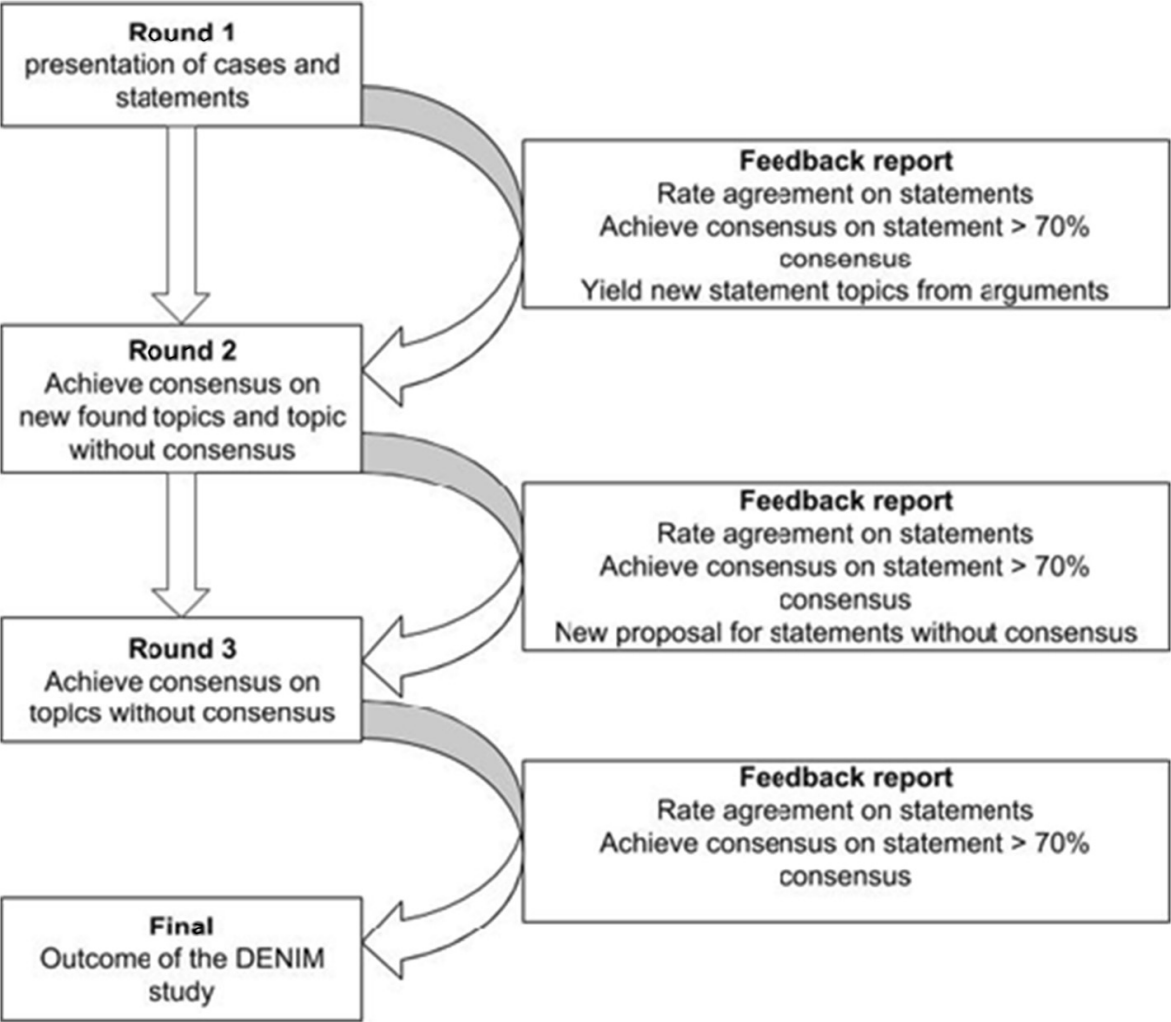
**The Delphi procedure for the DENIM study; rounds comprise of digital questionnaires, feedback report: is a supplemented file to the subsequent questionnaire, consensus: when at least 70% of all respondents agree with the statement, non-consensus: when at least 70% of all respondents disagree with the statement; DENIM: Delphi study in the Netherlands on the dispatch of the Mobile Medical Team.**

The DENIM study is structured as a three round digital Delphi procedure (Figure 2). In the first round the mean question is; which trauma patient deserves the advanced care of the mobile medical team? In order to generate discussion varying statements and cases will be introduced to the panel in the first round. The answers will be used to identify topics of interest leading to statements that will be tested in the subsequent round. Statements will be tested for level of agreement on a Likert scale. Moreover, distinctive concepts will be presented to the panellists, for instance *scoop and run, stay and play* or the use of neurological scales. Panellists were asked what description they thought best suited the concept or how they would assess the patient’s condition using different scales. For all questions panellists will be asked to motivate their opinion in an obligated open comment box before they could proceed, in pre text it is stated that the motivation to their answers is of critical importance for the subsequent round of the Delphi.

### Delphi questionnaires

The steering committee will develop the questionnaire for the first round. A list of themes and ideas of interest was constructed and mandatory topics were identified (Table 2). The questionnaires of all rounds will be designed and distributed using the online survey program SurveyMonkey®. In the first questionnaire, the main priority is to yield arguments and motivation. Three types of questions will be presented to the panellists (Table 3). Cases will be presented and panellists will be asked if they think that MMT care is indicated. Furthermore, open questions regarding definitions in trauma care and/or the type of treatment that is preferred in specific situations will be presented. Moreover, panellists will be asked to if they agree or disagree with statements that are presented. Considerations supporting their opinion should be stated after each question. Answers will be analysed using descriptive statistics and a sum of at least 70% of the experts that either totally agreed or agreed will be considered agreement as well as for disagreement. The first round will be assessed by an independent physician for feasibility and duration of undertaking the questionnaire before sending it to the panellists. In round two, a selection of questions derived from round one on the topics with no agreement nor disagreement will be retested using questions that elaborate on the subject. Furthermore, new topics will be introduced that have derived from the argumentation and considerations of the panellists and will likewise be tested.

**Table 2:** **List of relevant themes for the steering committee meeting**

**Themes prior to SC meeting**	**Additional topics after SC meeting**
1. Responsibility	1. What is a poly trauma patient
2. SitRap/MIST	2. Vital parameters/physiology
3. Expertise/Exposure	3. Patient characteristics
4. Logistics	4. Mechanism of trauma
5. Soft skills	5. Practical feasibility
6. Literature	6. Current dispatch criteria
7. Reason of dispatch	7. Communication
8. Regional differences	8. Surgical interventions
9. Prehospital judgment of o.a. consciousness	
10. Advance analgesia	
11. Function of the MMT	
12. Overview of integrated care	
13. On-scene-time	

SC: steering committee.

**Table 3:** **Type of questions used in round one**

**Type of question**	**Topic a.o.**	**Answer**
Cases	MMT presence	‘Yes’, ‘neutral’ or ‘no’
Open questions	EMV/AVPU	Open text field
Statements	Treatment options, parameters, patient characteristics, judgment	5-point Likert scale, ranging from “I totally agree” to “I totally disagree”.

MMT: Mobile Medical Team, EMV: element of the Glasgow coma scale, AVPU: acronym for measurement of patient’s level of consciousness (alert, voice, pain, unresponsive).

### Feedback

After each round results and argumentations of the previous round will be fed back to all panellists in an anonymous report including results and all argumentations given. The argumentation and comments given by panellist will be used to construct the subsequent questionnaire by the steering committee. The feedback reports will be supplemented to the questionnaire of the next round. The answers and comments will be presented both quantitatively (the distribution and sum of the agreement and disagreement per question) and qualitatively (the argumentation and comments of the panellists per statement) as well as whether or not agreement has been reached.

## Results

The objective of the DENIM study is to reach expert consensus on the question which trauma patient deserves the care of a MMT. This consensus will provide recommendations with which MMT dispatch criteria can be invigorated. This may lead to a more efficient deployment of the MMT for trauma in the Netherlands.

## Discussion

A Delphi technique is used for this study because it allows for group consensus to be reached amongst experts on a complex issue [11]. Due to the complexity of the prehospital decision-making-process, it is not feasible to generate a “one-size-fits-all” model. However, consensus can help to develop practice guidelines (i.e. dispatch criteria) and leave enough space for a patient tailored approach by professionals. Our research question cannot be addressed utilizing prospective trials because of ethical issues since MMT trauma care has been institutionalised for decades. However a Delphi procedure is a suitable research method because it is designed as an iterative process to combine expert opinion into group consensus [11]. It easily solicits the opinion from dominant, geographically dispersed and time poor experts, which is often the case with MMT-personnel, ambulance staff and trauma surgeons. One could debate that the Delphi does not correctly represents expert opinion as it is not a strict scientific untenable approach [17]. For instance, because the Delphi procedure does not use a random sample for selecting panellists. Therefore one has to ensure an accurate representation of the target population through a thorough selection process of respondents. To overcome this dilemma, criteria for qualitative studies are applied to help ensure credible interpretations of the findings. These criteria are based on the pillars of qualitative research such as; credibility, applicability and conformability [18]. We create ‘safety in numbers’ and the heterogeneity of the working background of the included panellists. This because panels comprising of similarly trained experts provide an effective and reliable utilization even of a small sample of experts and have proven to be a good base for the development of informed and effective decision-making criteria [19]. These decisions are strengthened by the utilization of reasoned arguments and assumptions that are challenged within this Delphi through feedback [20-22]. Feedback can be presented as a statistical group response, such as a measure of variance, along with that of central tendency of group opinion, accompanied by argumentations and comments provided by individual panellists [23]. Furthermore, it has been stated that results of a Delphi procedure are weakened because it does not allow discussion amongst experts directly [24]. However, the anonymous nature of the Delphi allows for a reduction of the biasing effects of dominant individuals in group-based discussion processes [14,25], especially in a hierarchal environment such as the Dutch healthcare system. It is essential that the validity of consensus depends on a sufficient response rate throughout all three iterations thus preventing a reduction in the quality of the information generated [26].

## Abbreviations

EMS: Emergency medical services
ATLS: Advanced Trauma Life Support
MMT: Mobile Medical Team
HEMS: Helicopter Emergency Medical Services
RIVM: The National Institute for Public Health and the Environment
DENIM: *Delphi studie in Nederland naar de Inzet van het MMT’,* Dutch acronym for: Delphi study in the Netherlands on the dispatch of the Mobile Medical Team
